# Surface Modifications and Their Effects on Titanium Dental Implants

**DOI:** 10.1155/2015/791725

**Published:** 2015-09-07

**Authors:** A. Jemat, M. J. Ghazali, M. Razali, Y. Otsuka

**Affiliations:** ^1^Department of Mechanical & Materials Engineering, Faculty of Engineering and Built Environment, UKM, 43600 Bangi, Selangor Darul Ehsan, Malaysia; ^2^Department of Peridontology, Faculty of Dentistry, National University of Malaysia, Jalan Raja Muda Abdul Aziz, 50300 Kuala Lumpur, Malaysia; ^3^Department of System Safety, Nagaoka University of Technology, 1603-1 Kamitomioka-Cho, Nagaoka-shi, Niigata 940-2188, Japan

## Abstract

This review covers several basic methodologies of surface treatment and their effects on titanium (Ti) implants. The importance of each treatment and its effects will be discussed in detail in order to compare their effectiveness in promoting osseointegration. Published literature for the last 18 years was selected with the use of keywords like titanium dental implant, surface roughness, coating, and osseointegration. Significant surface roughness played an important role in providing effective surface for bone implant contact, cell proliferation, and removal torque, despite having good mechanical properties. Overall, published studies indicated that an acid etched surface-modified and a coating application on commercial pure titanium implant was most preferable in producing the good surface roughness. Thus, a combination of a good surface roughness and mechanical properties of titanium could lead to successful dental implants.

## 1. Introduction

Surface treatments are normally carried out to modify yet maintain desirable properties of the substrate materials especially in the dental implant industry. The surface area can be increased remarkably by using proper modification techniques, either by addition or subtraction procedures [1, 2]. A surface treatment can also be classified into mechanical, chemical, and physical methods. In dental implant, the surface treatment is used to modify the surface topography and surface energy, resulting in an improved wettability [3–5], increased cell proliferation and growth [3], and accelerated osseointegration process [6]. The quality of dental implant depends on the properties of the surface. In order to have good interaction of the tissue and osseointegration, materials' biocompatibility and roughness of the surface played an important role. Goyal and coworkers [7] observed that the increased roughness can simultaneously increase the surface area of the implant, improve cell migration and attachment to implant, and enhance osseointegration process. Past literature has revealed most of the surface treatments able to brings a good effect to the dental implants [3–6]. Coating is proved to increase the surface area of the implants substantially [8]. The surface treated with plasma sprayed titanium exhibits the highest value of the surface roughness (3.43 ± 0.63 *μ*m) compared to machined surface (0.15 ± 0.04 *μ*m) [9]. The healing period was enhanced with hydroxyapatite (HA) coating compared to untreated one [10]. The behavior of modified surface on cells culture studies has revealed that an acid etched zirconia implant surface shows a significant improvement in cell proliferation, except for bone attachment and adhesion on the first day of culture [11–13]. In the study by Parsikia et al. [14], the commercially pure titanium surface was blasted followed by two-step chemical treatment (acid-alkali) resulting in optimized surface topography. The cell bioactivity was improved and expected to have good osseointegration at early stage. Furthermore, a rougher titanium surface promotes shorter healing process [15] than the smoother surfaces. Thus, the surface treatment is used not only to maintain the existing properties of the implants but also to enhance several behaviours as required by dental applications particularly in improving the healing process.

## 2. Background

### 2.1. Titanium Implant

Titanium is the material of choice for dental implant as its properties met the most important requirements such as excellent biocompatibility [16], corrosion resistance, high strength, and relatively low modulus of elasticity [17], good formability, and machinability. Additionally, surface modifications are being utilised on implant surfaces, mainly to improve wettability, cell-implant adhesion and attachment, cell proliferation, and osseointegration, and thus faster healing and shorter treatment duration. As a result, many research works have been carried out to improve surface modifications on existing implants to achieve the desired biological responses. The surface topography has also been manipulated such as acid etching and blasting [18] onto the surface to get a better topographies which consequently bring better roughness. In the case of the mechanisms, the roughness of the titanium implants was considered to be one of the significant parameters that affect the rate and the quality of osseointegration [15, 18, 19].

### 2.2. Biocompatibility of Titanium and Its Alloys

**Figure 1:**
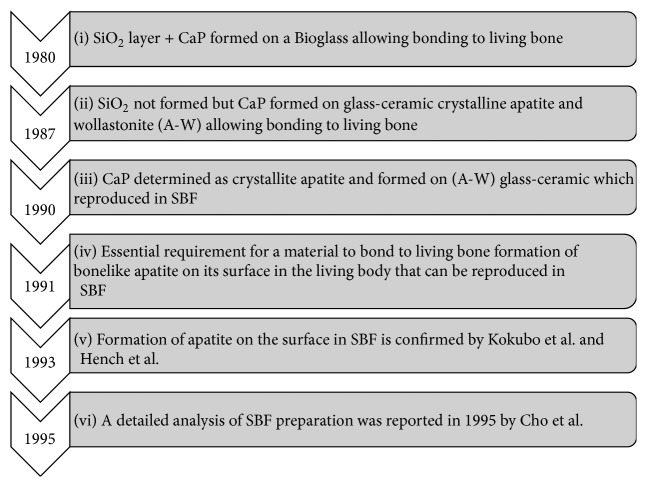
History of simulated body fluid (SBF).

Materials compatibility is the most important issue to be considered for a successful dental implantation. Titanium and its alloys are well known as materials that are well tolerated by living tissues and capable of promoting osseointegration [20]. Ideally, the modification of the implant surface was proposed to enhance osseointegration between materials and bone tissue. The surfaces of materials after treatment should be able to interact with the surrounding tissue to induce direct contact of bone to implant. Kokubo treatment, also known as simulated body fluid (SBF), is a chemical method for inducing or determining a level of biocompatibilities property of dental materials that was established in 1991 [21]. SBF can be described as a solution with ion concentration similar to human blood plasma (see Table 1), kept under mild conditions of pH and identical physiological temperature [21]. The history of SBF usage for apatite formation is shown in Figure 1 [21–25]. In early 1980, Ogino and coworkers [22] have found silicon dioxide (SiO_2_) layer and calcium phosphate (CaP) formed on a Bioglass which allows bonding to living bone. In 1990, Kokubo et al. [24] have stated that the formation of apatite is an essential for osseointegration between implant surface and living bone. The full preparation of SBF has been reported in 1995 by Cho et al. [25].

*In vivo* and* in vitro* bioactivity of a material can be predicted from the apatite formation on its surface in SBF [26]. Surface conditions, such as surface roughness, surface charge, surface energy, and chemical composition, have important influences on the osseointegration process. Therefore, modifying titanium implant surface seems to be a promising way to achieve stronger and faster osseointegration of the implants and also promoted shorter healing times from implant placement to restoration [27].

### 2.3. Surface Treatment

Recently, many works have been carried out on surface treated commercial titanium implants to enhance the osseointegration function (references). By increasing the surface roughness, an increase in the osseointegration rate and the biomechanical fixation of titanium implants have been observed [27, 28]. The implant modifications can be achieved either by additive or subtractive methods. The additive methods employed the treatment in which other materials are added to the surface, either superficial or integrated, categorized into coating and impregnation, respectively. While impregnation implies that the material/chemical agent is fully integrated into the titanium core, such as calcium phosphate crystals within TiO_2_ layer or incorporation of fluoride ions to surface, the coating on the other hand is addition of material/agent of various thicknesses superficially on the surface of core material. The coating techniques can include titanium plasma spraying (TPS), plasma sprayed hydroxyapatite (HA) coating, alumina coating, and biomimetic calcium phosphate (CaP) coating. Meanwhile, the subtractive techniques are the procedure to either remove the layer of core material or plastically deform the superficial surface and thus roughen the surface of core material. The common subtractive techniques are large-grit sands or ceramic particle blasts, acid etch, and anodization [19]. The removal of surface material by mechanical methods involved shaping/removing, grinding, machining, or grit blasting via physical force. A chemical treatment, either by using acids or using alkali solution of titanium alloys in particular, is normally performed not just to alter the surface roughness but also to modify the composition and to induce the wettability or the surface energy of the surface [29]. As for physical treatment such as plasma spray or thermal spray, it is often carried out on the outer coating surface to improve the aesthetic of the material and its performance. Additionally, ion implantation, laser treatment and sputtering [10, 30–33], alkali/acid etching [34–36], and ion deposition [37] are also utilised. Thus, in the light of studying the effects of surface treatments, this review only focuses on various methods that have high potentials in improving the performance of titanium implants. The basic principle of each surface modification and its developments are discussed in the following sections:

- Pretreatment significance.
- Plasma spray coating.
- Grit blasting.
- Acid etching.
- Dual acid etching (DAE).
- Sand blast and acid etching (SLA).
- Other methods.
- Trends in surface treatment of titanium.
- Final remarks.

#### 2.3.1. Pretreatment Significance

**Figure 2:**
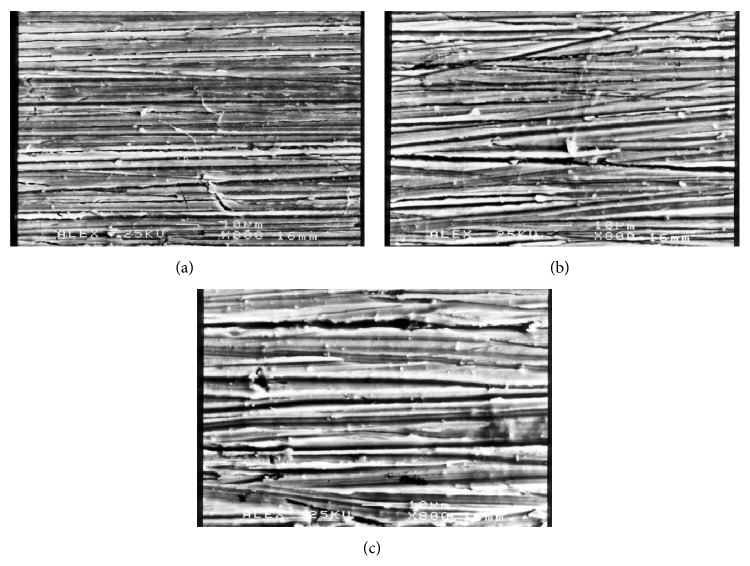
Typical morphologies of Ti alloy polished using SiC paper (a) 1200 grit, (b) 600 grit, and (c) 180 grit [42].

Prior to the surface modification, pretreatment is required to ensure the substrate surfaces are free from contaminations. Prior to plasma spray procedure, the substrates are normally pretreated by grit blasting [38, 39] to remove the surface impurities and roughened (roughness range 3–5 *μ*m) the surface in order to get better adhesion between substrate and powder [40]. The substrate can also be preheated to reduce residual stress and to avoid crack in the coating [38]. As for an acid etching method, the surface was prepared by polishing with several grits of sand papers [41] to achieve uniform [42] and regular morphology of the surface [43]. Typical surface roughness that is obtained from polishing process is in the range of ~0.1 [44, 45] to 3 *μ*m [43].  Figure 2 shows the typical morphologies of Ti alloy polished using silicon carbide (SiC) grit papers. In short, a pretreatment process is crucial as it provides clean surface, by eliminating undesired defects like scratch and irregularities.

## 3. Type of Surface Treatment

### 3.1. Plasma Spray Coating

**Figure 3:**
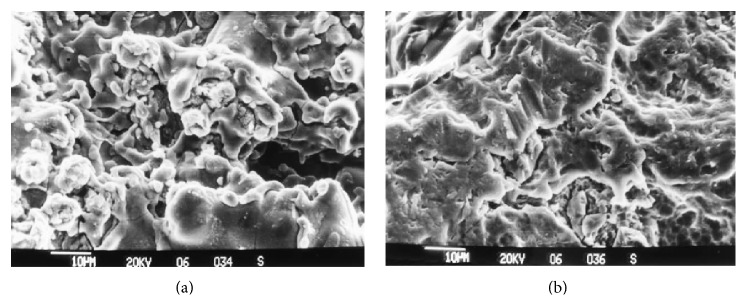
Surface morphology by (a) plasma sprayed titanium (b) deep profile structure [9].

Plasma spraying technique generally involves thick layer of depositions, such as hydroxyapatite (HA) and titanium (Ti). The coating process includes spraying thermally melted materials on the implant substrates. A combination of HA coating on Ti alloys substrate has received many attentions due to their attractive properties such as good biocompatibility and mechanical properties [32]. The plasma spray substantially increased the surface area of the implants by increasing their surface roughness [46]. The potential of spray plasma spray coatings to enhance the mechanical behaviour has been addressed by many studies [9, 17, 18, 31, 37, 38]. Several techniques were proposed to adhere HA to titanium implants [9, 10, 17], but only the plasma spraying coating technique has been successfully used on commercial implants [19]. A metastable calcium phosphate solution provides excellent bioactivity of the HA/YSZ/Ti-6Al-4V composite coatings, which have the ability to induce bone-like apatite nucleation and growth on implant surface [38]. Fouda et al. [10] reported that HA coated titanium implant could enhance the healing period compared to the uncoated implants. Xie et al. [33] also discovered that HA coatings promote better cell proliferation. However, in some cases, a reverse effect of HA coatings [47, 48] was also noted. According to Liu et al. [49], the bonding strength of HA on titanium alloys decreased long hours of immersion time in the simulated body fluids (SBF). Yang et al. [48] also reported that after an immersion in the SBF, the hydroxyapatite (HA) coatings became weak due to the intermellar or cohesive bonding degradation in the coating. However, Knabe et al. [9] found that a plasma sprayed titanium surface exhibits the highest surface roughness compared to a deep profile surface structure (the surface was acid etched and grit blasted; see Figure 3) and in an* in vitro* test, the HA coating has less bone contact compared to other surface modifications. Some reports showed that the mechanical properties of HA can be significantly improved by the addition of yttria-stabilized zirconia [40, 50]. Previous study [51] reported that the HA coatings reinforced with zirconia possessed better performance in bond strength and dissolution behaviour of the titanium implants. Over the same period (4 weeks after the SBF immersion), the HA/YSZ/Ti-6Al-4V composite coating showed a reduced tensile strength by ~27.7% compared to the pure HA coatings with ~78.8% [38]. It has been reported that more new bones are formed and grow more rapidly into pores of the surface of alkaline-modified plasma sprayed implants, and this may be beneficial to reduce clinical healing times and thus to improve implant success rates [52].

### 3.2. Grit Blasting

Another route for roughening the surface is grit blasting, through pressurised particle projection either using ceramic materials or silica onto the implant surface. Materials such as sand, hydroxyapatite, alumina, or TiO_2_ particles are usually employed for the purposes [35, 36]. Grit blasting is always followed by an acid etching to remove the residual blasting particles. Hence, the grit blasting is also considered as one of the means to embed surface contaminants on the substrates [51]. Surface microhardness of zirconia particles on titanium surface via blasting was found to be far greater than a controlled polished titanium surface [19]. Al-Radha and coworkers [53] evaluated the effect of bacterial adhesion on several titanium implants with different treatments. The results showed that ZrO_2_-blasted titanium exhibited greater bacterial adhesion compared to other surface treatments. In a similar case, Aparicio et al. [34] applied alumina blasting with particle sizes ranging 425–600 *μ*m to gain high value of surface roughness between 4.15 ± 0.26 *μ*m. In* in vivo* studies by Bacchelli et al. [54], they discovered that deposited titanium treated with commercially pure Ti shows the highest surface roughness of 8.55 ± 0.78 *μ*m, followed by ZrO_2_ sandblasting with improved osteogenesis. This indicates that the blasting method also has an effective role in inducing optimum roughness of dental implants surface [3]. However, this technique is only promising in a good surface but not in terms of osseointegration itself. Besides, bacteria will tend to accumulate more on the rough surface substrate compared to smooth substrate. Thus, further study on how this technique affects the important properties like bone implant contact, removal torque values, tissues response, and bacterial adhesion, and biocompatibility must be carried out.

### 3.3. Acid Etching

In acid etching, the use of acids on metal surfaces is not only to clean the surface but also to modify the roughness. A strong acid like hydrofluoric (HF), nitric (HNO_3_), and sulphuric (H_2_SO_4_) or a combination of these acids is commonly used in this technique. Acid etched surfaces had increased cell adhesion and bone formation, thus enhancing the osseointegration [3, 49–51, 53, 54, 59–62]. Due to its dissolution ability [63, 64], HF has been used for etching restorative ceramic materials in order to increase the bonding surface for luting agents. The significance of this technique also renders the substrate with homogeneous roughening regardless of the sizes and shapes [63]. The roughness of titanium is one of the factors that helps in determining the stability of bone formation and resorption at the interface of bone implants [65]. Alla et al. [66] reported that a nanotopography that allows bone ingrowth via acid etching on an implant may improve the roughness. Previous study has reported that the rate of etching depends on the type and concentration of the acid used [35]. However, the suitability of these acids in etching was not determined as they required further tests particularly on the bone implant contact and torque removal. Titanium samples etched by H_2_SO_4_ with different concentrations demonstrated an increase in surface roughness [57]. Concentrated H_2_SO_4_ has been proven as an effective solution to roughen the surfaces particularly for biological applications [66].

#### 3.3.1. Dual Acid Etching (DAE)

**Figure 4:**
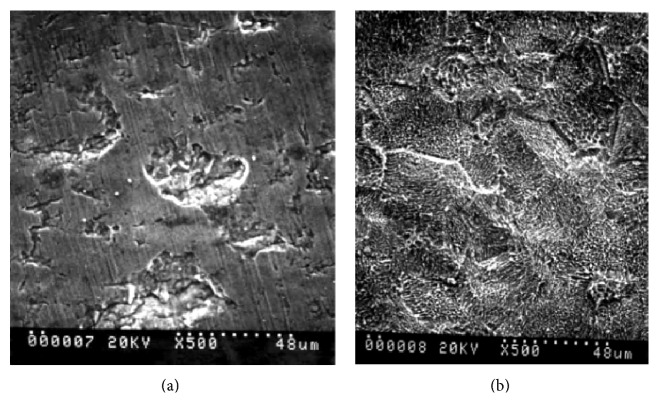
Titanium implant with (a) a machined surface and (b) treated dual acid 48% HF + HCl/H_2_SO_4_ [51].

Similar to acid etching, the DAE is also able to treat the surface via chemical or acid whether in sequence [45] or with the combination of both [67, 68]. Rapid osseointegration can be achieved by dual etching through micro rough surface [55]. A comparative study between a machined surface and those using HF and HCl/H_2_SO_4_ (DAE) has shown the acid treated surface has greater resistance to reverse torque removal and better osseointegration [55]. In order to examine the surface roughened by the DAE, Yang et al. [48] inserted fifteen implants into rabbit's tibias. It was remarkable to note that roughened surfaces implants showed greater value of a removal torque at 2, 4, and 8 weeks than the machined surface. At the same time, a histomorphometric analysis demonstrated that the bone-to-implant contact significantly increased along with the peri implant bone formation. Thus, the DAE can provide a surface with a certain microroughness, thus contributing to a rapid osseointegration [35]. However, the acid etching treatment is strongly dependent on the acid selection and the process. Juodzbalys et al. [69] observed that an acid etched titanium implant exhibited similar surface topography as those gained from a sand-blasted large-grit acid etched (SLA) surface treatment. They found that the sample of titanium shows a good surface roughness with 1–10 *μ*m micropits after etching with H_2_SO_4_ and then HCl compared to a poor surface microtexture by HCl and then H_2_S0_4_ [69]. A comparison study had also been carried out between a machined surface and a dual-etched surface as shown in Figure 4.

It was noted that the acid treated surface gave greater resistance in a reverse torque removal and better osseointegration than the machined surface implants [55]. A surface treatment via acid etching on zirconia implant has been reported to have similar effects on density of the bone implant and relative capacity for an osseointegration [61]. However, side effects like porosities, with sizes ranging from 0.5 to 2 *μ*m, were also formed due to the use of these acids [58, 70]. This process somehow is also believed to benefit tissue ingrowth and cell surface interactions in the dental implant [70]. The success of osseointegration or implant anchorage was measured using a resistance to reverse torque rotation. As torque rotation force value increased, bone-to-implant contact (BIC) also increased which lead to greater osseointegration [51].

### 3.4. Sandblast, Large-Grit, and Acid Etching (SLA)

**Figure 5:**
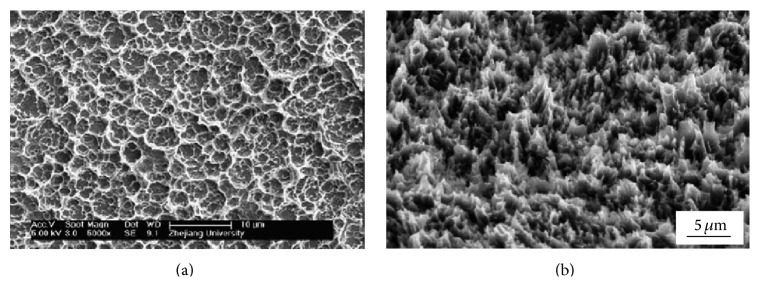
The surface morphology of (a) sandblasted and treated Ti6Al4V alloy implants with DAE (HCl and H_2_SO_4_) [57] and (b) sandblasted and etched Ti implant with warm HCl [58].

SLA is used to induce surface erosion by applying a strong acid onto the blasted surface [17]. This treatment combines blasting with large-grit sand particles and acid etching sequentially to obtain macro roughness and micro pits [58] to increase the surface roughness as well as osseointegration [71–74]. Cho and Jung [71] discovered that the SLA surface possessed wide cavities (from 5 *μ*m to 20 *μ*m in diameter) and micro pits (from ~0.5 *μ*m to 3 *μ*m in diameter), indicating an increase in the surface roughness and the surface area. Hence, the SLA-treated surface was found to be useful for improving tissue integration and cell proliferation.* In vivo* studies on six adult dogs carried out by Xue et al. [52] indicated that the surface after sequential grit blasting and alkaline treatment showed high shear strength, improving early bone growth and osseointegration. A recent investigation on a two-step chemical treatment (acid-alkali) noticed that optimised morphology and good bioactivity resulted in good osseointegration during the early stage of the implantation [75]. Similarly, He et al. [76] also discovered that the implants treated with blasting followed by the DAE (HCl and H_2_SO_4_) promote better osseointegration during the healing phase, indicating a great improvement in the bioactivities. In addition, biological evaluation by Kim et al. [58] discovered that human osteoblasts grow splendidly on the SLA surface which provides greater space for cell attachment and proliferation. Surface morphology for SLA typically became rough and irregular after sandblasting, but then after the acid etching treatment the surface is more uniform and small micro pits (1-2 *μ*m in diameter) are created as shown in Figures 5(a) and 5(b).

### 3.5. Other Methods

Ion implantation, laser treatment, sputtering, and other combinations of several mentioned techniques are also briefly discussed in this review. An ion implantation, for example, involved accelerating ions of materials in an electrical field and impact onto the substrate to a depth of approximately 1 *μ*m [1]. Braceras and his colleagues [37] used this method to investigate the osseointegration properties of the treated implant surface. They found out that the ion implantation of cobalt onto titanium alloys significantly improved the osseointegration. Deposition via dip coating of nanocomposite (HA-ZrO_2_-Al_2_O_3_) on titanium substrate showed the highest adhesion strength compared to the HA coatings [37]. Another technique observed by Pető et al. [77] involve Nd glass laser, in which the removal torque of implants was 20% larger for laser-treated surface compared to the machined and blasted implants. These results corresponded well with the data reported by Hallgren et al. [75] who demonstrated that the removal torque value was larger for the laser-modified implant (52 Ncm) than the machined surface implant (35 Ncm) after 12 weeks of healing. This result was also in agreement with other studies [9, 78–81]. Using pulsed magnetron sputtering method [72], ZrO_2_-Ag and ZrO_2_-Cu deposited titanium surface had improved the antibacterial performance relative to pure Ti implant materials [58]. In another study, combination method of laser-treated and acid etched surface was proven to have better osseointegration than the laser-treated surface with BIC value 49.71% [80].

## 4. Trends in Surface Treatment of Titanium

**Figure 6:**
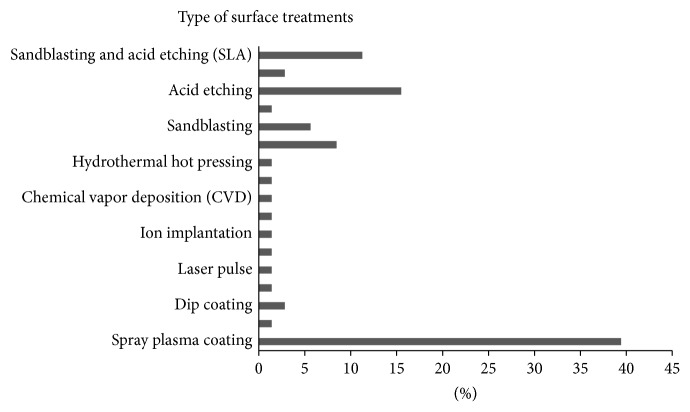
Surface treatments commonly used in titanium dental implants.

The greatest interest has been noted in the use of plasma spraying and acid etching techniques. Clearly, the plasma spray method is the most preferable (see Figure 6) due to its advantages in providing porous implant surfaces for greater bone contact [30]. The qualities of the coating surface are strongly dependent on the types of the coating materials. Other than that, study on plasma spray showed good growth cells on the implant surface [9] and a good bone contact which accelerated the bone formation [30]. Relatively, coated implants like ZrO_2_ possessed high surface roughness with an approximation of 5.7 ± 0.2 *μ*m. This value could be increased up to 8.68 ± 0.37 *μ*m [47] when acid etching is applied prior to coating. Even the dual acid etching played an important role in producing good surfaces, with roughness ranging from 0.44 to 3.51 *μ*m [34, 35, 82]. In general, the DAE is better than a single acid etching due to its high composition, amount, and concentration. In the case of dental implants, the effect of acid etching is based on the concentration and the type of the acid as well as the temperature and time, in which the surface roughness normally increased with an increasing concentration of acid [67]. Furthermore, the blasting and the SLA techniques were also commonly used to improve the surface roughness and have high potential to improve the implant bone healing. Every single technique has its own advantages and limitations. Thus, to ensure high quality of the coated materials, the importance of a pretreatment on the surface prior to depositing work must also be considered.

In this paper, important methodologies that have been extensively utilised in the surface treatment work of titanium dental implants are summarised in Table 2. In spite of the high number on plasma spray method of studies to date, the results in the literature demonstrate difficulties in deciding the optimum value of surface roughness for better osseointegration yet decrease bacterial adhesion.

## 5. Final Remarks

All in all, the coating techniques contribute to important positive effects of dental implant application. Most authors [6, 9, 30, 31, 33, 56, 79–81, 83–86] agreed that a good coating technique may give high impact on the mechanical properties of the dental implants. However, this technique has several limitations including poor long-term adherence of the coating to the substrate material [76], nonuniformity in thickness of the deposited layer [84], and variations in crystallinity [87] and composition of the coating. On the practical side, a better understanding of the suitable parameters during plasma spray is important in order to control these limitations. In contrast, most studies could not determine any major advantages or disadvantages with blasted surface implants. Blasting is one popular technique for surface treatments which can easily roughen the implant surface but is inadequate to give credit to the important properties like bone implant contact, removal torque values, tissues response, and biocompatibilities. Ion implantation technique on the other hand is useful to harden the surface of titanium but not applicable for dental implant [64]. It is most useful in orthopaedic devices which are subject to articulating or in wear situations. Another preferable surface treatment technique is the DAE that has high composition, amount, and concentration [63]. To date, ceramic coatings (calcium phosphate, HA, and TiO_2_) still remain the most popular bioceramic materials in the surface treatments area. Nevertheless, HA is recognised as the best candidate in bioceramics compared to TiO_2_ [76]. Meanwhile, zirconia also has good potential as dental implants whereby it promotes higher microhardness [33] and better mechanical properties when coated onto Ti alloy. Zirconia stabilized with yttria (YSZ) particles as a secondary phase in coatings is also believed to be dispersion-strengthened due to the homogeneous distribution of YSZ particles in the matrix [84], resulting in good bonding within the composite, and hence improves the mechanical properties.

Currently, surface roughening (e.g., grit blasting, acid etching, and SLA) and coating (e.g., with CaP and HA) are commonly used techniques in practice. Both methods have their advantages and drawbacks as we have discussed in this paper. It has been reported that the improvement of bone implant interface and greater resistance of failure were influenced by acid etched surface [45]. In addition, sandblasted with large-grit (25–50 mm) and acid etched surface were found to have a 50–60% mean value of bone implant contact compared to titanium plasma sprayed surface which had only a 30–40% mean value of bone implant contact after 6 weeks [46]. BIC value is very important in long-term success of dental implants. Numerous studies have demonstrated that rough implants surface show better bone apposition and BIC than implants with smooth surfaces [22, 46, 75]. Surface roughness also stimulated the cell migration and proliferation which in turn leads to better BIC [50]. Different modification methods have been studied, namely, sandblasted, large-grit, acid etched (SLA) and coated surfaces that were chemically different but had the same physical properties that were conducted to assess BIC as a measure of osseointegration.

It is clearly noted that by altering or modifying the surface texture, namely, the roughness of titanium implants, in particular, desired effects can be obtained like bone implant contact, removal torque values, tissues response, and biocompatibility. Thus, most works still favour surface treatment of dental implants via coating and acid etching over other methods in producing good substrate surfaces for osseointegration, with surface roughness ranging from 0.44 to 8.68 *μ*m. In short, a good surface with the right roughness and mechanical properties could lead to better osseointegration for successful dental implants.

## Figures and Tables

**Table 1:** Ion concentrations (mM) of SBF and human blood plasma [21].

Ion	Simulated body fluid (SBF)	Blood plasma
Na^+^	142.0	142.0
K^+^	5.0	5.0
Mg^2+^	1.5	1.5
Ca^2+^	2.5	2.5
Cl^−^	148.8	103.0
HCO^3−^	4.2	27.0
HPO_4_^2−^	1.0	1.0
SO_4_^2−^	0.5	0.5

**Table 2:** Studies on the surface treatment on Ti dental implants.

Source(s)	Ti type	Surface treatment	Findings	Average roughness Ra (*µ*m)
Knabe et al. [9]	CP-Ti ASTM-F67	Plasma spray Ti coating, acid etching, and sandblasting	All implants except HA coating surface showed good growth cells.	Ti coating 3.43 ± 0.63
		HA coating		HA coating 2.07 ± 0.36

Depprich et al. [11]	ZrO_2_	Acid etching	Acid etched surface shows similar properties of osseointegration with titanium implant.	0.598
	Ti	Acid etching		1.77

Hung et al. [17]	CP-Ti (Ti-6Al-4V ELI, ASTM-F136)	Plasma sprayed hydroxyapatite (HA)	Treated implants indicate high biocompatibility for bone regeneration of titanium implants.	Sa 9.36

Eom et al. [18]	Ti	(1) Blasting HA	Hybrid type coating shows higher bone implant contact and removal torque value (259.9 ± 6.2 Ncm) than other surfaces.	1.2–1.8
		(2) Blasting and dual acid etching (SLA)		2.5–3.0
		(3) hybrid-type coating with HA and blasting		3.0–3.5

Darimont et al. [30]	CP-Ti	HA coating Titanium plasma sprayed	HA coating exhibited higher value of bone contact and accelerated the formation of bone.	NR

Simmons et al. [32]	CP-Ti	Sintered porous surface Ti spray plasma	The adhesion properties of the porous surface implants are more stiffer and stronger than plasma sprayed implants	NR

Xie et al. [33]	CP-Ti	Plasma sprayed dicalcium silicate/ ZrO_2_	Higher ZrO_2_ content coating layer exhibits smaller dissolution and lesser degree of degradation.	NR

Aparicio et al. [34]	CP-Ti ASTM B348	(1) Acid etching	Blasted and alkaline etched plus thermal formed rough and bioactive surface lead to accelerate bone tissue regeneration and increased mechanical retention in the bone.	1.69 ± 0.1
		(2) Grit blasting		4.74 ± 0.2
		(3) Grit blasted and alkaline etched + thermos chemical treatment		4.23 ± 0.2

Ban et al. [35]	CP-Ti	Acid etching with variable parameter (temperature and time)	Surface roughness increased as temperature and time increased. Weight loss increased linearly with time and temperature.	0.44–3.51

Velasco-Ortega et al. [36]	CP-Ti	Sandblasting with alumina and nitric acid etching (SLA)	After surface treatment, cpTi implant achieved high biocompatibility with no cytotoxic.	NR

Yang et al. [48]	Ti	YSZ plasma spray Acid etching	After acid etching, the Ti surface is roughened and may enhance the osseointegration.	8.68 ± 0.37

Al-Radha et al. [53]	Ti	(1) Blasting with ZrO_2_	Blasted ZrO_2_ surface showed a very good effect on adhesion reducing almost similar to pure ZrO_2_ properties.	0.158 ± 0.003
		(2) Blasting with ZrO_2 _and acid etching (SLA)		0.150 ± 0.005

Chou and Chang [55]	Ti	Grit blasting with alumina and then ZrO_2_ sprayed plasma	ZrO_2_ bond coat promotes adhesion mechanism for Ti substrate.	NR

Simon et al. [56]	cpTi	Ti plasma spray	Surface roughness by Ti coating may optimize the osseointegration and enhance the clinical function.	4.4 ± 0.37

NR = No Result.
